# Unveiling the Mystery: A Case Report on Oropharyngeal Synovial Sarcoma

**DOI:** 10.7759/cureus.44703

**Published:** 2023-09-05

**Authors:** Soraia Gonçalves, Catarina Soares, Rita Ribeiro, Sílvia Sousa

**Affiliations:** 1 Family Medicine, Unidade Local de Saúde do Alto Minho, Viana do Castelo, PRT

**Keywords:** head and neck synovial sarcoma, oropharyngeal mass, case report, clinical case report, family medicine

## Abstract

Synovial sarcoma, originating from mesenchymal cells, represents a rare and aggressive sarcoma subtype.

This case report describes a rare occurrence of synovial sarcoma in the soft palate, with only a few cases described in the literature.

A 38-year-old male presented with a painless mass on the soft palate, which raised suspicion of an abscess and emphasized the importance of considering malignancy in persistent or progressive soft tissue masses, even in atypical anatomical locations. The diagnostic workup, including imaging modalities such as maxillofacial computed tomography (CT) scan, magnetic resonance imaging (MRI), and positron emission tomography-fluorodeoxyglucose (PET-FDG) scan, played a crucial role in confirming the diagnosis and assessing disease extension.

The standard treatment is the complete excision of the tumor. Nevertheless, when it comes to tumors located in the head and neck region, defining standardized margins proves to be a challenge. Radiotherapy can play an important role, particularly in those with tumors larger than 5 cm or positive margins. While chemotherapy offers certain advantages, its application remains a subject of controversy despite its potential benefits.

Timely referral and multidisciplinary management are essential in optimizing patient outcomes. Although synovial sarcoma poses diagnostic and therapeutic challenges, advances in diagnostic techniques and personalized medicine offer hope for improved outcomes.

## Introduction

Originating from mesenchymal cells, sarcomas represent a rare and heterogeneous group of malignant tumors. They account for less than 1% of all adult cancers [1,2]. The most common subtypes are liposarcoma, leiomyosarcoma, undifferentiated pleomorphic sarcoma, and gastrointestinal stromal tumors [3]. Concerning synovial sarcoma, the cell of its origin remains unknown, and it can be classified into two morphological subtypes: monophasic and biphasic [4].

Although most cases of sarcoma lack a clearly defined etiology, several associated or predisposing factors have been identified. Alongside well-known genetic predisposition syndromes like Li-Fraumeni syndrome and neurofibromatoses type I, there is a growing recognition that a significant proportion of sarcoma patients may carry pathogenic germline variants. Other predisposing factors include retinoblastoma, exposure to radiation therapy, chemotherapy, chemical carcinogens, chronic irritation, lymphedema, as well as the involvement of human immunodeficiency virus (HIV) and human herpes virus 8 in the pathogenesis of Kaposi sarcoma [5]. Lifestyle factors usually linked to cancer, such as smoking, diet, or exercise are not considered risk factors for soft tissue sarcoma [6].

Typically, sarcoma presents as a painless, gradually growing soft tissue tumor in young adults [7]. In rare cases, patients may exhibit constitutional symptoms, such as fever or unexplained weight loss [8]. In fact, regarding synovial sarcomas, the mean duration of symptoms before the correct diagnosis is approximately two years. However, considering the head and neck the diagnosis was accomplished 20 months earlier, representing a four-month gap between the appearance of the first symptoms and the diagnosis [9]. While sarcoma can develop in any anatomical part, the majority of them involve the extremities, with only 9% occurring in the head and neck region. When considering the oral cavity, the majority of reported cases affect the jaw bones and tongue [7]. According to Kadapa et al., only about ten cases were described in the literature until 2014 [7].

The differential diagnosis includes benign soft tissue tumors, such as lipoma, as well as malignant tumors, such as sarcoma, metastatic carcinoma, melanoma, or lymphoma. The United Kingdom Department of Health has published criteria for soft tissue lesions that deserve an urgent referral: a soft tissue mass with a diameter superior to 5 cm, a painful lump, a lump that is increasing in size, a lump of any size that is deep to the muscle fascia, or recurrence of a lump after previous excision [10].

In terms of treatment, further investigation is necessary. The standard treatment is the complete excision of the tumor; however, there are no standard margins defined for head and neck sarcomas. Radiotherapy can also play an important role in the treatment, especially in the postoperative treatment when the patient presents with positive or marginal margins or tumors larger than 5 cm. Nevertheless, the radiation dose is not yet standardized [11]. Regarding chemotherapy, it seems to be a survival benefit; however, its effectiveness remains a topic of controversy [12].

This clinical case, due to the rarity of oropharyngeal synovial sarcoma, evident in its scarcity in medical literature, emphasizes a crucial point: the necessity to alert clinicians about its unique clinical presentation, diagnostic challenges, and the urgency of early referral. This rarity underscores the need for heightened clinical suspicion, timely assessment, and appropriate management, all of which can profoundly impact patient outcomes and survival rates. 

## Case presentation

A 38-year-old male presented to his family doctor in August 2022, with a painless mass on the right side of the soft palate, with three weeks of evolution. He worked as a constructor and did not have any relevant medical history, regular medication, allergies, or history of smoking and drinking habits. He had no fever or any other associated symptoms. On physical examination, the mass was approximately 1 cm in diameter when observed, with intact mucosa, mild signs of inflammation, and a soft consistency upon palpation. The patient was apyretic, had a blood pressure of 133/81 mmHg, a heart rate of 82 bpm, and a body mass index of 22 kg/m^2^. Due to its presentation as a bulging of the posterior soft palate with mild inflammatory signs, at that time, it was interpreted as a peritonsillar abscess. The patient received treatment with Amoxicillin + Clavulanic acid (875 mg + 125 mg) every 12 hours for eight days, in addition to topical (diclofenac 0.074% oral solution, 15 cc, three times a day) and systemic anti-inflammatory (ibuprofen 600 mg twice a day for five days) medications.

**Figure 1:**
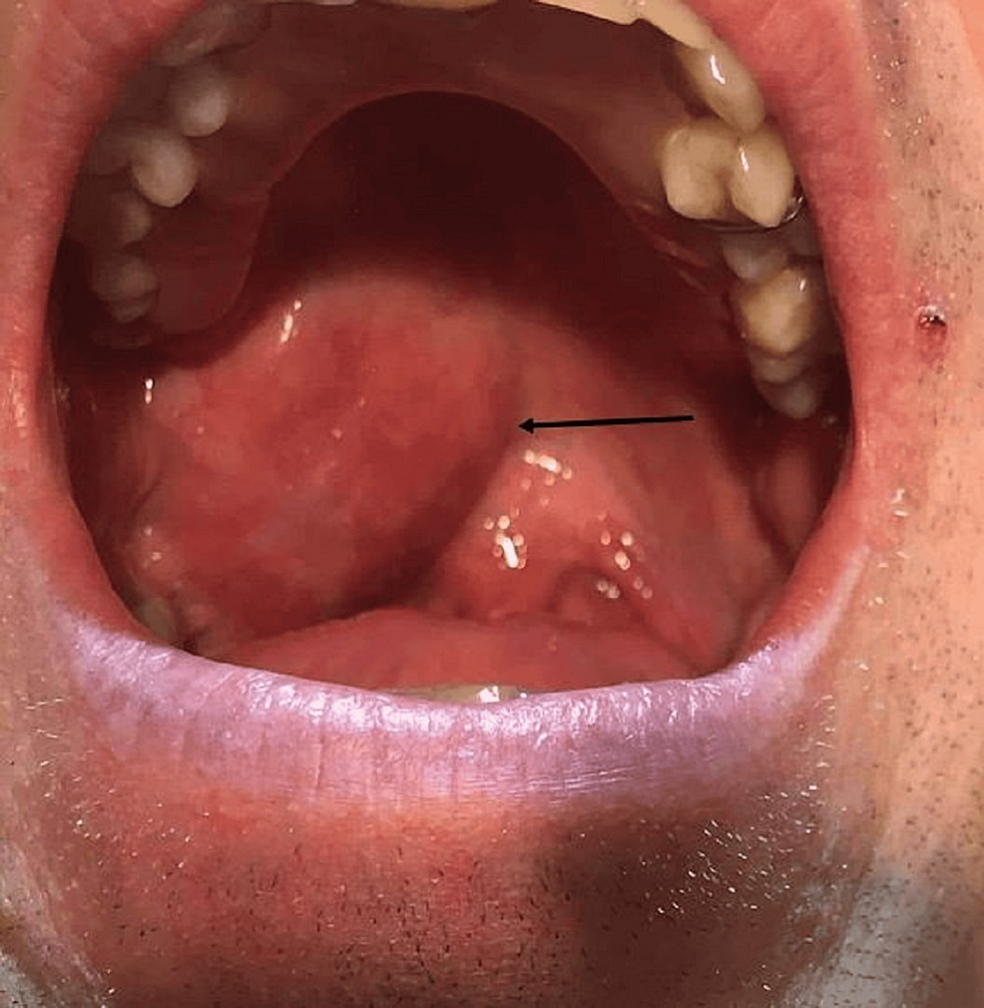
Oropharyngeal mass. Mass at the time the patient was sent to the emergency room.

A follow-up appointment was scheduled for reevaluation a week later. Due to the lack of improvement, an increase in the size of the lesion, and new complaints of intermittent dyspnea and odynophagia within five days, he was referred to the local hospital's emergency department on that day (Figure 1).

**Figure 2:**
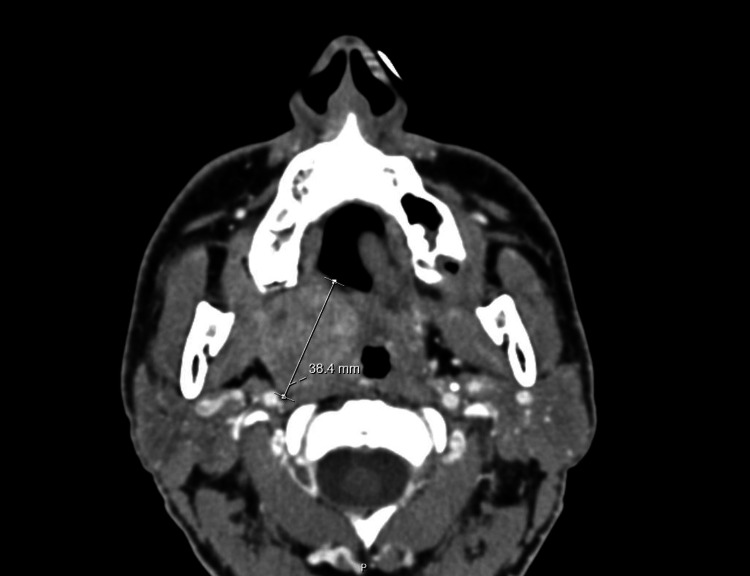
CT scan: transverse plane (mass with approximately 39 mm). CT, computed tomography

**Figure 3:**
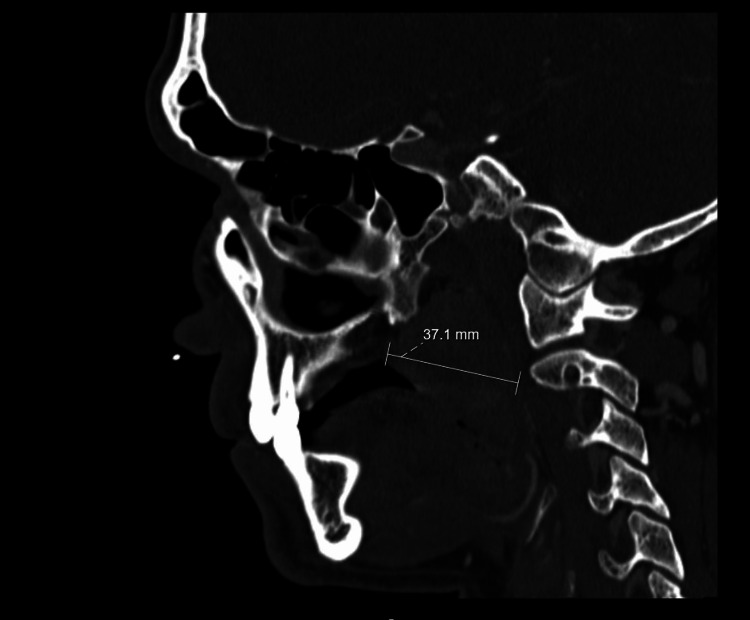
CT scan: sagittal plane (mass with approximately 37 mm). CT, computed tomography

At the emergency department, blood tests showed no significant changes and a maxillofacial CT scan revealed a large expansive lesion centered on the right palatine tonsil, well-circumscribed, hypodense, and heterogeneously enhancing, measuring approximately 39 mm in maximum diameter. The possibility of a granuloma mimicking an abscess should be considered by clinical and laboratory data. The alternative of a tumor should only be considered based on objective observation. Nodular formations with a diameter of less than 10 mm, with a hypodense central component, were also present in both superior internal jugular chains, which were likely to be adenitis. These are likely to be adenitis, although reactive lymph nodes with typical bilateral central hilum coexist. No other significant pathological changes were observed (Figures 2-3) Due to the following findings, the patient was subsequently referred to the Otorhinolaryngology (ORL) department of the nearest hospital.

Following the initial assessment by the ORL department, surgical excision was scheduled for three weeks later, at the end of September.

**Figure 4:**
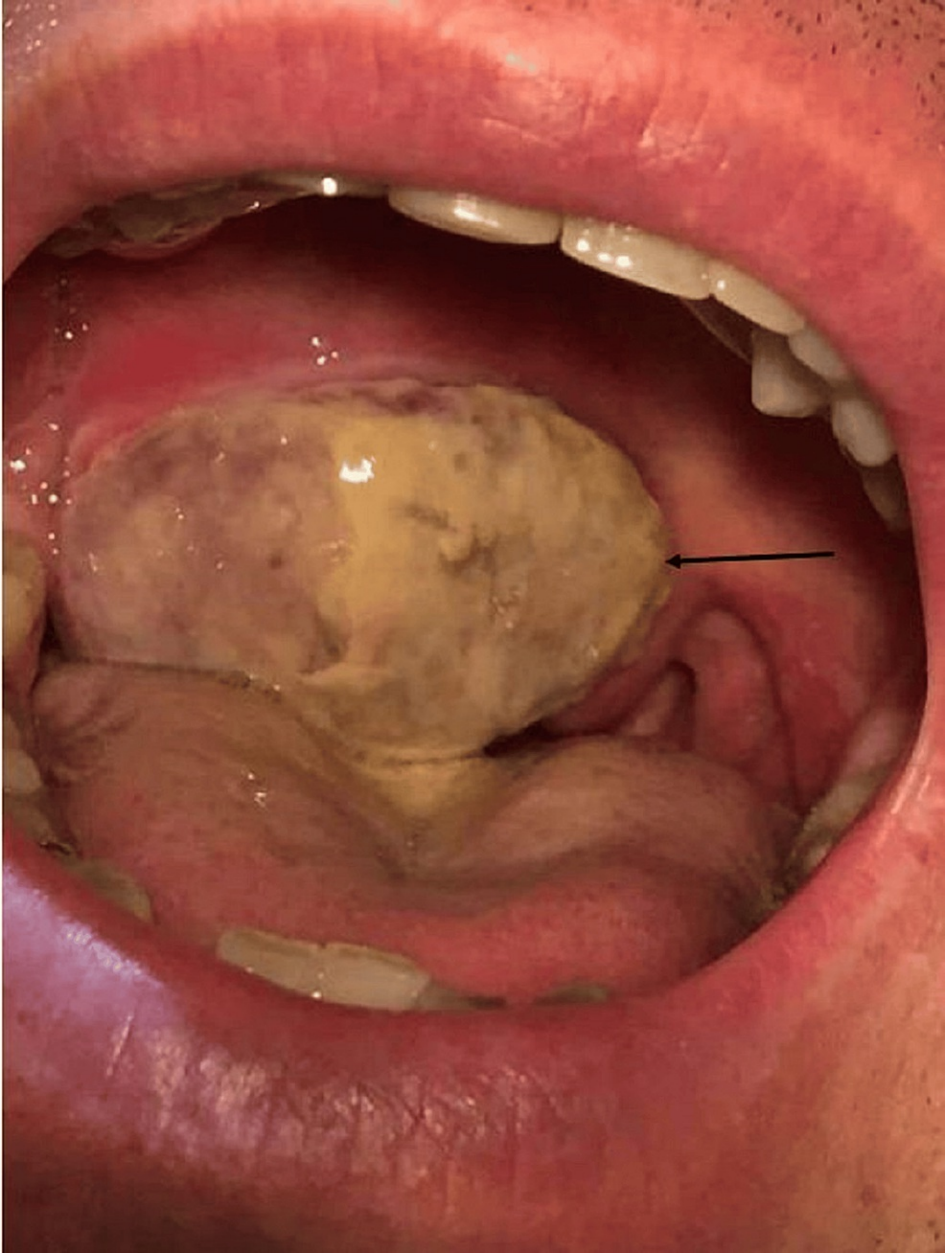
The mass after the partial surgical excision.

Two months after the first appointment, in October 2022, after the partial surgical excision, during a follow-up ORL appointment, it was observed that the mass had increased in size. At this time, it had breached the barrier of the soft palate, had a considerably more suspicious appearance, was covered with fibrin, and had a vegetative aspect. The patient was then treated with tramadol, metoclopramide, and metronidazole, and magnetic resonance imaging (MRI) and positron emission tomography-fluorodeoxyglucose (PET-FDG) scans were requested. Four days later, the patient returned to the emergency department due to further enlargement of the mass. On physical examination, a lesion occupying the soft palate was observed, covered with fibrin, with a vegetative appearance, extending beyond the midline and touching the tongue (Figure 4). It did not seem to reach the palatine tonsil. The patient was discharged with corticosteroid therapy.

In mid-October, the pathology results revealed Synovial Sarcoma of Biphasic Subtype (FNCLCC score 3). The patient underwent a PET-FDG scan, which suggested active malignant neoplasm. Bilateral superior lateral cervical lymph node formations, with short-axis measurements in the subcentimeter range, some showing very slight uptake, making it impossible to exclude the possibility of being lymph node lesions. Additionally, a CT-SPN scan report stated: "Compared to the previous maxillofacial CT scan, there is an increase in the size of the known expansive formation centered on the right/paramedian right aspect of the soft palate, currently extending to the uvula, which appears edematous and expanded. (...) Conclusion: Given the histological result of synovial sarcoma, the staging is considered T3 N0."

The patient was then referred to the Portuguese Oncology Institute for consultation with the sarcoma group. The patient underwent neoadjuvant chemotherapy, starting at the end of October 2022. After five cycles of chemotherapy, in March 2023, the patient underwent surgery with a right paramedian mandibulotomy approach, extensive resection with conservative cervical lymph node dissection (levels I to III on the right side), and reconstruction using a free left forearm flap.

Currently, in July 2023, the patient has been proposed for adjuvant radiotherapy due to the findings on the pathology report, which revealed a synovial sarcoma with unclear margins.

## Discussion

Synovial sarcoma is a rare and aggressive subtype of sarcoma originating from mesenchymal cells [1,10]. While it can arise in various anatomical locations, its occurrence in the soft palate is extremely rare, with only a few reported cases in the literature [7,11].

The patient's initial presentation featured a painless mass on the posterior soft palate, initially raising suspicion of a peritonsillar abscess due to the short duration of symptoms, mild inflammatory signs, and the absence of constitutional symptoms. While symptoms like odynophagia are often associated with peritonsillar abscess, their absence is not definitive [13]. As reported in the literature, the most frequent complaint of head and neck synovial sarcoma is the appearance of a painless mass [2,7]. Nevertheless, given its higher frequency and the characteristics previously mentioned, an abscess was considered the primary diagnosis. Despite the initial diagnosis, the atypical presentation should alert the importance of an early reevaluation. In this case, at the reevaluation, it was noticed an increasing mass size, without any signs of improvement and new symptoms such as dyspnea and odynophagia. These findings prompted the patient's referral to the emergency room (ER), leading to a workup that allowed for the diagnosis to be anticipated by three months, compared to the literature [9]. Considering the diagnosis of sarcomas, a delay in diagnosis is frequent due to their rarity and diverse clinical presentations, as shown in the clinical case by Algargaz et al. [10]. 

This highlights the importance of considering malignancy in the differential diagnosis of persistent or progressive soft tissue masses, even in atypical locations.

In terms of treatment, in the present case, after the histological confirmation of diagnosis, the patient underwent five cycles of chemotherapy followed by surgery with extensive resection and conservative cervical lymph node dissection. As the surgical histological report revealed positive margins, adjuvant radiotherapy was proposed. In the literature, the standard treatment is the complete excision of the tumor with radiotherapy playing an important role in the presence of positive or marginal margins [11]. Nevertheless, as there are no standard margins or radiation dose, different approaches have been performed, as described by Kouhen et al., where the two presented cases underwent surgery followed by radiotherapy with different radiation doses, and neither of the patients received chemotherapy treatment [9]. This enhances the importance of further investigation to define standardized treatment.

As family doctors, maintaining an astute awareness of uncommon differential diagnoses is paramount in identifying and referring patients to specialized care promptly. This is particularly evident in the presented case, where the patient's medical history did not overtly suggest a predisposition to synovial sarcoma. However, exploring potential underlying genetic predispositions or environmental exposures that could contribute to sarcoma development remains essential.

The diagnostic journey was significantly aided by advanced imaging modalities such as maxillofacial CT scan, MRI, and PET-FDG scans. These imaging techniques played a pivotal role in characterizing the lesion, gauging its extent, and evaluating the potential presence of metastatic disease [14]. Swift referral of the patient proved pivotal for timely treatment initiation. Subsequent follow-up and surveillance are vital not only for monitoring disease recurrence but also for managing potential late effects of treatment.

Patient-centered care is a core commitment for family doctors, enabling comprehensive and seamless management. Longitudinal follow-up fosters reassessment and early detection of changes. This approach aligns with holistic patient needs, ensuring continuous monitoring.

The profound impact of heightened awareness around synovial sarcoma's varied presentations, including rare sites like the soft palate, is paramount. This awareness revolutionizes patient outcomes through early detection and accurate diagnosis - the cornerstones of effective management. This case underscores how embracing heightened awareness mitigates delays, optimizes care, and improves prognosis in synovial sarcoma cases.

By synergizing healthcare provider vigilance, patient-centered care, and a commitment to increased awareness, the medical community can profoundly influence the trajectory of challenging cases like synovial sarcoma, underscoring the potential for positive transformation in patient care and outcomes.

## Conclusions

We presented a rare case of synovial sarcoma in the soft palate, emphasizing the significance of considering malignancy in the differential diagnosis of soft tissue masses. A multidisciplinary approach is essential to ensure accurate diagnosis and appropriate management, with early recognition and timely intervention playing a critical role in improving patient outcomes.

To further enhance our understanding and management of this challenging malignancy, continued research and collaboration are essential.
